# Profiling the Fatty Acids Content of Ornamental *Camellia* Seeds Cultivated in Galicia by an Optimized Matrix Solid-Phase Dispersion Extraction

**DOI:** 10.3390/bioengineering4040087

**Published:** 2017-10-17

**Authors:** Carmen Garcia-Jares, Marta Sanchez-Nande, Juan Pablo Lamas, Marta Lores

**Affiliations:** Laboratorio de Investigación y Desarrollo de Soluciones Analíticas (LIDSA), Departamento de Química Analítica, Nutrición y Bromatología, Universidade de Santiago de Compostela, Facultad de Quimica, Avda das Ciencias s/n, Campus Vida, E-15782 Santiago de Compostela, Spain; martasancheznande@gmail.com (M.S.-N.); juanpablo.lamas@usc.es (J.P.L.)

**Keywords:** *Camellia* seeds, fatty acids, limonene, matrix solid-phase dispersion, GC-MS

## Abstract

*Camellia* (genus of flowering plants of fam. *Theaceae*) is one of the main crops in Asia, where tea and oil from leaves and seeds have been utilized for thousands of years. This plant is excellently adapted to the climate and soil of Galicia (northwestern Spain) and northern Portugal where it is grown not only as an ornamental plant, but to be evaluated as a source of bioactive compounds. In this work, the main fatty acids were extracted from *Camellia* seeds of four varieties of *Camellia*: *sasanqua*, *reticulata*, *japonica* and *sinensis*, by means of matrix-solid phase dispersion (MSPD), and analyzed by gas chromatography (GC) with MS detection of the corresponding methyl esters. MSPD constitutes an efficient and greener alternative to conventional extraction techniques, moreover if it is combined with the use of green solvents such as limonene. The optimization of the MSPD extraction procedure has been conducted using a multivariate approach based on strategies of experimental design, which enabled the simultaneous evaluation of the factors influencing the extraction efficiency as well as interactions between factors. The optimized method was applied to characterize the fatty acids profiles of four *Camellia* varieties seeds, allowing us to compare their fatty acid composition.

## 1. Introduction

The genus *Camellia* (*Theales*, *Theaceae*) is native from Asia and comprises different species cultivated worldwide for different uses and applications. Among them are *Camellia sinensis* (the tea plant), and the flowered species *C. japonica*, *C. reticulata*, and *C. sasanqua*, mostly cultivated as ornamental plants [1]. The main species used for oil production is *Camellia oleifera*, also known as oil-tea camellia. However, the seeds of other *Camellia* species also contain remarkable fatty acids, with a high proportion of oleic and linoleic acids and low saturated acids, this general lipidic profile being associated to well- known healthy properties [2].

In Spain, the commercial production of camellias is located mainly in Galicia, a northwestern region where camellias, mostly cultivars of *C. japonica*, have been cultivated in parks and gardens for over 200 years and currently more than 30 nurseries produce 2.5 million camellia plants annually [1]. In addition, many plants from other *Camellia* species are also widely distributed in this geographic area [3]. In this context, the evaluation of the fatty acid profile of the seeds of the most common species in this region is considered with the final goal of obtaining extracts with potential applications in the cosmetic and food industries.

The selection of the extraction method influences the physicochemical characteristics of the extracts obtained [4]. The traditional methods for recovering oil from seeds are hydraulic pressing, expeller pressing, and organic solvent extraction [5]. Besides, most commercial camellia seed oil is obtained by either hexane extraction or by a process that combines expeller pressing and hexane extraction [5]. However, hexane extraction has some undesirable properties, such as flammability, possible contamination of the oil by solvent residues, and air pollution, entailing safety and environmental inconveniences [6]. Therefore, the trend in the extraction of bioactive compounds is using solvents classified as “generally recognized as safe” (GRAS) and food grade solvents. Among them, limonene constitutes an interesting solvent for the extraction of fatty acids from natural sources [7]. (+)-Limonene is a renewable chemical, by-product of the citrus industry, with growing applications in the food and cosmetic industry [8], and is generally considered safe.

From an analytical point of view, Soxhlet has been one of the most conventional methods of extraction used to extract vegetable seeds although it requires high extraction times and solvent consumption [9,10,11]. To avoid these kinds of drawbacks, alternative, greener extraction procedures have been applied to *Camellia*, including supercritical fluid extraction [9,12,13], microwaves-assisted extraction [14], and salt effect-aided aqueous extraction [15].

Since its introduction in 1989 [16], matrix solid phase dispersion (MSPD) has been demonstrated to be an efficient technique for the isolation of a wide range of compounds from a wide variety of complex samples. MSPD is based on several simple principles of chemistry and physics, involving forces applied to the sample by mechanical blending to produce complete sample disruption and the interactions of the sample matrix with a solid support bonded-phase or the surface chemistry of other solid support materials [17].

The analysis of fatty acids has been mostly performed by GC after derivatization to their methyl esters, using conventional detectors such as FID [9,11,18], and MS detectors [10,19]. The use of HPLC with evaporative light scattering detector has also been described for the analysis of the underivatized acids [20], as well as other techniques including near infrared transmitance spectroscopy and pattern recognition techniques [21].

In this work, a MSPD method of extraction is optimized using multivariate experimental design strategies to get camellia extracts rich in fatty acids, and gas chromatography-mass spectrometry (GC-MS) is used to characterize the composition of the extracts. Among the studied factors, both dispersant and solvent are evaluated, considering both classical and green solvents with very interesting possibilities to the extraction of natural compounds [7]. In this work, *C. japonica* seeds are used for method optimization, and their fatty acids profile is then compared to the profiles of other three camellia varieties also grown in the Galicia region.

## 2. Experimental Section

### 2.1. Chemicals

Ethyl acetate and hexane were purchased from Sigma-Aldrich (Deisenhofen, Germany), limonene and ethyl lactate from SAFC-Sigma (Deisenhofen, Germany), and methyl-*tert*-butyl ether (MTBE) from Merck (Darmstadt, Germany). Dispersants were washed sea sand (200–300 μm, Scharlau), florisil^®^ from Supelco Analytical (Bellefonte, PA, USA), diatomaceous earth Hyflo^®^ Super Cel^®^, and C18-reversed phase silica gel (C18) from Sigma-Aldrich. Anhydrous sodium sulphate (Na_2_SO_4_, 99%) was purchased to Panreac (Barcelona, Spain), and the derivatizating agent trimethylsulfonium hydroxide (TMSH) was obtained from Sigma-Aldrich. All solvents and reagents were of analytical grade. MSPD extraction was performed in commercially available 15 mL plastic cartridges. Polyethylene filter frits (IST, 16 mm/20 μm, Supelco, Deisenhofen, Germany) were used to compact the clean-up phase and the dispersed sample in the MSPD column.

Pure fatty acids standards (97–99%) were all supplied by Sigma-Aldrich (St. Louis, MO, USA) namely: palmitic, *cis*- and *trans*-oleic, linoleic, linolenic and stearic acids. Individual standard stock solutions of 10 mg mL^−1^ were prepared in MTBE. Working solutions were obtained by appropriate dilution. Solutions were stored at −20 °C protected from light.

### 2.2. Samples

Samples were obtained from different plant nurseries of the A Coruña province (Galicia, Spain): *C. japonica* from Viveros Orvihouse, *C. sasanqua* and *C. sinensis* from Viveros Brandariz, and *C. reticulata* from Cooperativa Estelas da Terra. The fruits were collected in September 2015, and introduced in hermetic plastic bags (50 × 30 cm). Then they were transferred to plastic boxes with paper until the fruits became opened, and in a period of one week the seeds were collected and stored in zip plastic bags (20 × 20 cm) at room temperature. To express the results in dry weight (dw), the moisture content of the samples was calculated. For that, 3 g of seeds were dried in an oven at 105 °C and weighed before and after the dryness step. This operation was carried out in triplicate.

### 2.3. MSPD

**Figure 1:**
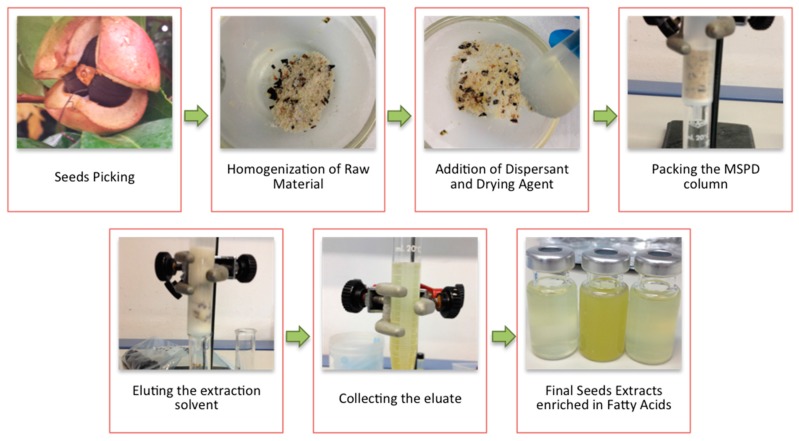
Scheme of the Matrix Solid Phase Dispersion Procedure for *Camellia* Seeds.

Seeds and dispersant (sand or florisil) were weighed, introduced into a glass mortar (ratio 1:4), and grinded to obtain a homogeneous mixture. This step favored the rupture of the seed tissues and the subsequent extraction of the fatty acids. Since the average humidity of the seed samples was 18.7%, 2 g of anhydrous sodium sulphate were added as drying agent, also facilitating the dispersion of the sample. The mixture was transferred into a MSPD column provided with a polypropylene frit and filled with 1 g of washed sea sand at the bottom. A second frit was placed on top of the dispersed sample before compression with a syringe plunger. Elution was made by gravity flow using the selected solvent and the selected elution volume for each particular experiment. The eluate was collected into a graduated conical tube (Figure 1), transferred to 10 mL vials and stored at −20 °C until the GC-MS analysis. It could be noticed that crushed seeds in the mortar gave off an unpleasant odor, also noticeable in the extracts. Using limonene as the extraction solvent, the bad smell of the seeds was eliminated.

### 2.4. Gas Chromatography-Mass Spectrometry

The MSPD extracts contain the original fatty acids from the seed samples. Before analysis by GC-MS, fatty acids are usually transformed into their less polar methyl ester derivatives (FAMEs) by derivatization. In this work, trimethylsulfonium hydroxide (TMSH) was chosen as the derivatization reagent, based on our previous studies [22]. In brief, 100 μL of the seeds extract were evaporated under gentle nitrogen stream. Then, the residue was dissolved in 500 μL MTBE, and 100 μL of this solution was placed in a screw-cap vial containing 50 μL TMSH. This was vortexed for 30 s, and allowed to react for 30 min. Then, the sample is ready for GC-MS analysis. The GC-MS analysis of the FAME extracts was performed with an Agilent7890A (GC)-Agilent 5975C inert MSD with triple axis detector and an Agilent7693 autosampler from Agilent Technologies (Palo Alto, CA, USA), and a Zebron ZB-semivolatiles column (30 m × 0.25 mm × 0.25 μm). Helium (purity 99.999%) was used as the carrier gas, at a constant column flow rate of 1 mL min^−1^. The remaining chromatographic and MS conditions are summarized in Table 1.

To identify the FAMEs, the retention times and the mass spectra were compared with those obtained from the standards. FAMEs were quantified by an external standard calibration procedure in a concentration range from 1 µg mL^−1^ to 50 µg mL^−1^.

### 2.5. Statistical Analysis

Data analysis, regression, ANOVA and experimental design, were accomplished using Statgraphics Centurion XV (Manugistics, Rockville, MD, USA) as software package. A multifactor categorical design was applied for the optimization of the MSPD extraction procedure to analyze the simultaneous effect of the sorbent and the elution solvent, both nature and volume, affecting MSPD. The chromatographic peak area of each one of the target analytes was used as the selected response.

## 3. Results and Discussion

### 3.1. Optimization of the MSPD Extraction

MSPD selectivity strictly depends on both the nature of the sorbent materials and the elution solvent employed [16]. Thus, some preliminary experiments were firstly carried out in order to test the performance of a number of solvents and sorbents in the extraction of fatty acids from *Camellia* seeds by MSPD. Four solvents, namely hexane, ethyl acetate, ethyl lactate, and limonene; and four dispersants: florisil^®^, sea sand, C18, and diatomaceous earth, were studied. The obtained results showed that hexane, limonene, and ethyl acetate, enabled a sensitive and selective elution of the fatty acids, with the worst performance of ethyl lactate. Among dispersants, diatomaceous earth and C18 were discarded since they produced a very slow elution. Besides, C18 is costly and diatomaceous earth is very voluminous, complicating the maintenance of the prefixed sample:dispersant ratio (1:4). Then, dispersants selected for further optimization by experimental design were florisil and sand.

Therefore, the simultaneous evaluation on MSPD efficiency of the solvent nature (Factor A, three levels), the dispersive phase (Factor B, two levels), and the extraction volume (Factor C, two levels), was performed by a factorial design. The selected experimental domain is shown in Table 2 along with the values of the prefixed factors. A multifactor categorical design, involving 12 randomized experiments, was selected. The results can be easily interpreted with the statistical tools provided by the software. The analysis of variance (ANOVA) describes the effect of the studied factors on the obtained response (Table 3).

The *F*-ratio measures the contribution of each factor or interaction on the variance of the response. The *p*-value tests the statistical significance of each factor and interaction. In this table, it can be seen that the extraction of the main fatty acids form camellia seeds is independent of the nature of the selected solvent (A). On the contrary, both the dispersant phase and the second order factor (AB), meaning the interaction between dispersant and solvent, are statistically relevant. The dispersant (B) was significant at the 95% confidence level (*p* value < 0.05) for two of the analytes, and the interaction AB for three of them, excluding the stearic acid. The eluting volume (C) was not significant for any of the target compounds.

**Figure 2:**
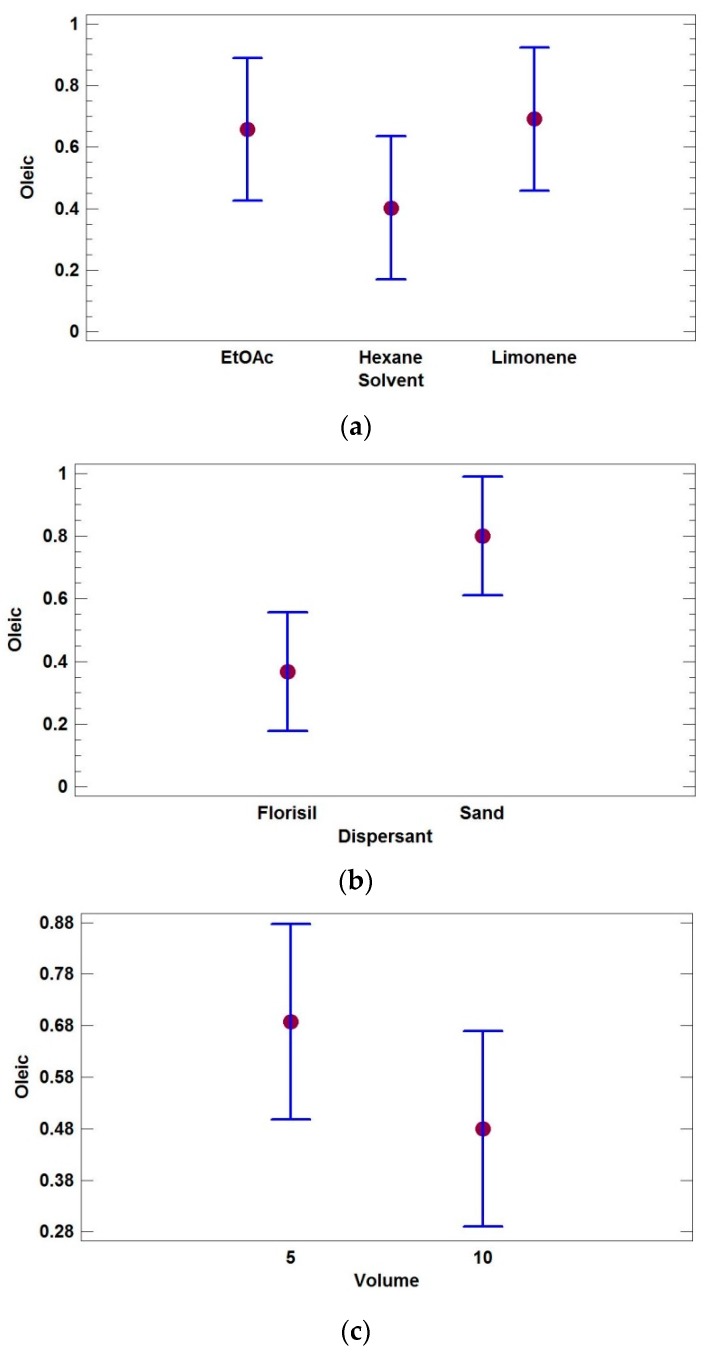
Mean plots of the main factors studied in the multi-factor categorical design for oleic acid: (**a**) extraction solvent; (**b**) dispersant type; (**c**) eluting volume.

Figure 2 shows the mean plots for one of the target acids (oleic), since all the fatty acids showed a similar behavior regarding the most suitable extraction conditions.

These plots (Figure 2) illustrate the effect of the variables by showing the mean values as well as the confidence intervals for each level (LSD at 95%), and can be employed to identify which level of each factor led to higher responses for a specific compound. Analyzing the mean plots for the extraction solvent (factor A), limonene was found to be the most suitable solvent, although in general, equivalent responses were obtained independently of the solvent used (Figure 2a). The sorbent type (factor B) was a statistically significant for two of the acids, with sand providing the most effective recoveries (see Figure 2b). Regarding the elution volume (factor C), no difference or a slight increase in the chromatographic signal was found when 5 mL instead of 10 mL of extract were collected, although this response improvement was not statistically significant for any of the targets (Figure 2c; Table 2).

**Figure 3:**
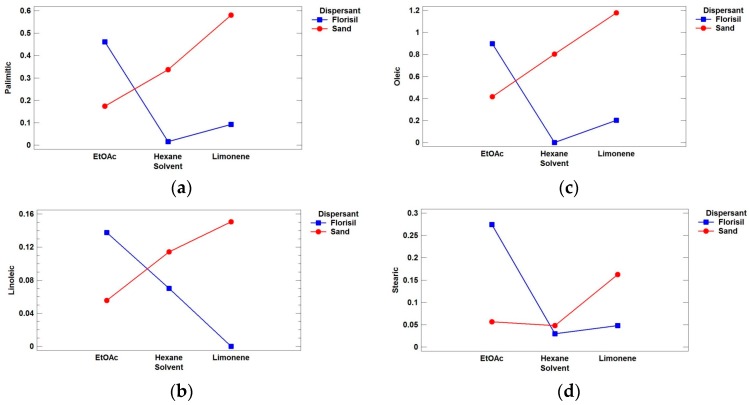
Interaction plots between the adsorbent type and the extraction solvent for the target fatty acids: (**a**) palmitic; (**b**) linoleic; (**c**) oleic; and (**d**) stearic.

From Table 2, it can be noticed that only one interaction was statistically significant, that between solvent and dispersant (AB). The interaction plots for the four acids are depicted in Figure 3, showing the average response at each combination of solvent (A) and dispersant (B).

A significant response improvement was observed for all the targets when the combination sand-limonene was used (Figure 3a–c), with the exception of stearic acid (Figure 3d). For this acid, the best combination was florisil-ethyl acetate, which was the second best for the other acids. Then, taking into account the results of the design as well as other considerations such as the price of the dispersant (sand is much cheaper than florisil), and the novelty and sustainability of limonene, this combination was selected as optimal for the extraction for the selected acids from *Camellia* seeds.

After optimization, the proposed MSPD procedure implied blending 1 g of *Camellia* seeds with 4 g of sand and 2 g of anhydrous Na_2_SO_4_, and elution with 10 mL of limonene.

### 3.2. Method Performance

Linearity of the GC-MS/MS method was evaluated in a range of 1–50 µg mL^−1^ considering six concentration levels and three replicates by level. Results are shown in Table 3. Determination coefficients (R^2^ ≥ 0.9916) demonstrated a directly proportional relationship between the amount of each analyte and the chromatographic response. Intra-day precision was evaluated at three concentration levels (5, 10 and 50 µg mL^−1^) and inter-day precision was evaluated at two concentrations (5 and 35 µg mL^−1^). Results are also shown in Table 4. In general, repeatability (intra-day, *n* = 3) as well as reproducibility (inter-day, *n* = 6), showed satisfactory values (RSD ≤ 10%) with the exception of linoleic acid at the lowest concentration (RSD = 23%). Instrumental limits of detection (IDL) were calculated as the concentration giving a signal-to-noise ≥3, and were <0.198 µg mL^−1^ (see Table 4).

Precision of the whole analytical procedure was evaluated by analyzing five independent extracts of Camellia japonica in two different days. Results showed good precision with RSD ≤ 8.20%.

### 3.3. Fatty Acids Profiles of Camellia Seeds Cultivated in Galicia

**Figure 4:**
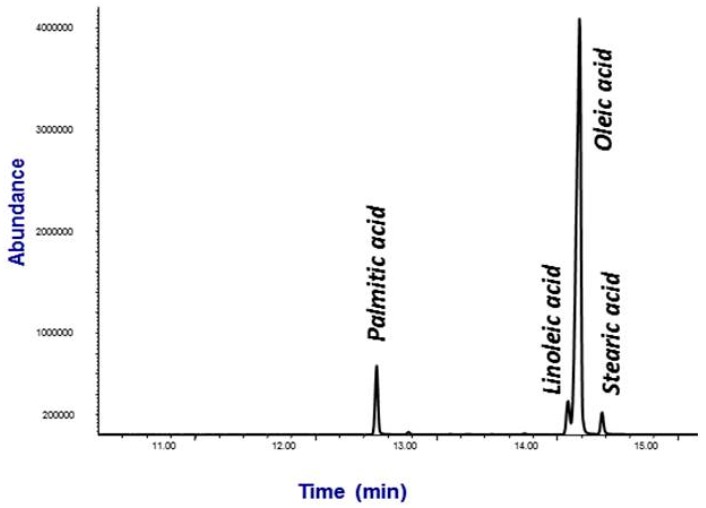
Total ion chromatogram (TIC) showing the main fatty acids of Camellia japonica seeds.

The method was applied to samples of four Camellia species: sasanqua, sinensis, japonica and reticulata, cultivated in Galicia. Figure 4 shows the total ion chromatogram (TIC) of a *C. japonica* extract sample. Four of the considered acids (palmitic, stearic, oleic, and linoleic) could be quantified in the seeds. Table 5 shows the fatty acids profile of each variety.

In terms of absolute amount, the extracts of *C. japonica* were the richest in fatty acids followed by *C. reticulata* and *C. sasanqua*. *C. sinensis* extracts contained much lower fatty acids amount. In *Camellia* extracts the content of unsaturated acids represented up to 92% of the total, which is comparable to virgin olive oil fatty acids composition, and is in agreement with previous findings [3]. Oleic acid was predominant, representing about 80% of the total fatty acid content, with the exception of *C. sinensis* (64%), in which it is noticeable the high linoleic acid proportion found in the seeds extracts (19%), as it was also reported by Wang et al. [9]. Saturated fatty acids were also important in *C. sinensis* seeds (17%), which was about the double than in the other varieties. These findings are in good agreement with those reported in a recent paper [11].

## 4. Conclusions

The fatty acids profiles of seeds from four varieties of *Camellia* grown in Galicia (northwestern Spain) as ornamental trees: *sasanqua*, *reticulata*, *japonica* and *sinensis*, were studied. The main acids were extracted using an optimized matrix-solid phase dispersion extraction method. GC-MS analysis of the corresponding methyl esters allowed us to characterize the fatty acids profiles of the four *Camellia* seeds, finding quite similar profiles for three of the varieties (*sasanqua*, *reticulata*, and *japonica*), in which oleic acid represented about 80% of the total, whereas *C. sinensis* showed an average of 64% of oleic acid, being the variety with the highest proportion of linoleic and stearic acids. The optimization of the MSPD extraction procedure by multivariate analysis enabled the simultaneous evaluation of the factors influencing the extraction efficiency and also factors interactions. The use of green solvents such as limonene constituted an efficient and greener alternative to conventional extraction solvents.

## Figures and Tables

**Table 1:** Gas chromatography-mass spectrometry (GC-MS) analytical parameters.

**Splitless Pulse Injection**	30 psi for 1.25 min
**Injection volume**	1 µL
**Injector temperature**	260 °C
**Column**	ZB-Semivolatiles (30 m × 0.25 mm × 0.25 μm)
**Temperature Program**	60 °C (1 min), 20 °C min^−1^ to 150 °C;
	10 °C min^−1^ to 290 °C (10.5 min)
	10 °C min^−1^ to 310 °C (3 min)
**Mass spectrometer**	Transfer line at 310 °C
	Ion source at 230 °C
	Quadrupole at 150 °C
**Mode**	Electronic Ionization (EI)
	Full Scan *m*/*z*: 45–450 amu

**Table 2:** Factors and levels considered in the experimental design.

**Factor**	***Code***	***Low Level***	***Central Level***	***High Level***	***Continuous***
Extraction Solvent	A	Ethyl acetate	Hexane	Limonene	No
Dispersive Phase	B	Sand		Florisil	No
Eluting Volume (mL)	C	5		10	Yes
**Fixed factors**	***Value***				
Sample Size	1 g				
Ratio Sample: Dispersant	1:4				
Drying agent	2 g				

**Table 3:** Analysis of variance (ANOVA) table showing the significance of main effects and second order interactions (*F* ratios and *p* values).

Acid	*A: Solvent*		*B: Dispersant*		*C: Volume*		*D: Interaction AB*	
	*F* Ratio	*p* Value	*F* Ratio	*p* Value	*F* Ratio	*p* Value	*F* Ratio	*p* Value
**Palmitic (C16:0)**	9.13	0.0987	27.01	**0.0351**	5.88	0.1362	49.75	**0.0197**
**Stearic (C18:0)**	2.97	0.2518	0.44	0.5742	0.09	0.7938	5.39	0.1564
**Oleic (C18:1)**	4.28	0.1896	24.20	**0.0389**	5.57	0.1422	27.20	**0.0355**
**Linoleic (C18:2)**	1.07	0.4836	8.84	0.0969	3.90	0.1868	28.51	**0.0339**

Values in bold denote statistical significance (*p* < 0.05).

**Table 4:** Linearity, instrumental limits of detection, and precision of the GC-MS/MS method (intra-day at 5, 10, and 50 µg mL^−1^, inter-day at 5 and 35 µg mL^−1^).

Acid	Linearity		IDL (µg mL^−1^)	Precision (RSD%)				
	Range (µg mL^−1^)	R^2^		Intra-Day (*n* = 3)			Inter-Day (*n* = 9)	
				5	10	50	5	35
C16:0	1–50	0.9929	0.198	5.09	2.45	2.55	3.05	3.13
C18:0	1–50	0.9916	0.056	3.23	7.63	3.75	7.12	3.15
Cis-C18:1	1–50	0.9922	0.051	10.4	3.00	2.14	5.40	9.99
Trans-C18:1	1–50	0.9921	0.100	6.19	1.15	1.06	7.14	2.88
C18:2	1–50	0.9960	0.135	23.0	4.39	1.15	14.0	8.67
C18:3	1–50	0.9926	0.123	2.98	0.77	2.83	2.61	1.89

**Table 5:** Fatty acids content (%) in seeds from Camellia grown in Galicia (SUA, sum of unsaturated fatty acids, SSA, sum of saturated fatty acids).

	*C. sasanqua*	*C. reticulata*	*C. sinensis*	*C. japonica*
C16:0	5.8 ± 0.9	6.7 ± 0.6	9.4 ± 0.0	7.5 ± 0.6
C18:0	1.8 ± 0.4	2.8 ± 0.1	7.5 ± 1.2	1.4 ± 0.4
C18:1	84 ± 2	78 ± 1	64 ± 0	82 ± 1
C18:2	8.7 ± 0.5	13 ± 0	19 ± 1	9.5 ± 0.4
SUA	92 ± 1	91 ± 1	83 ± 1	91 ± 1
SSA	7.6 ± 1.3	9.4 ± 0.7	17 ± 1	8.9 ± 0.7
